# The Effect of Berberine on Reproduction and Metabolism in Women with Polycystic Ovary Syndrome: A Systematic Review and Meta-Analysis of Randomized Control Trials

**DOI:** 10.1155/2019/7918631

**Published:** 2019-12-13

**Authors:** Liangzhen Xie, Duojia Zhang, Hongli Ma, Hui He, Qing Xia, Wenjuan Shen, Hui Chang, Yingying Deng, Qi Wu, Jing Cong, Chi Chiu Wang, Xiaoke Wu

**Affiliations:** ^1^First Affiliated Hospital, Heilongjiang University of Chinese Medicine, Harbin, China; ^2^Heilongjiang University of Chinese Medicine, Harbin, China; ^3^Taizhou Hospital of Chinese Medicine, Taizhou, China; ^4^Department of Obstetrics and Gynaecology, Prince of Wales Hospital, The Chinese University of Hong Kong, Shatin, Hong Kong

## Abstract

**Purpose:**

To assess the efficacy and safety of berberine on reproductive endocrine and metabolic outcomes in women with polycystic ovary syndrome (PCOS).

**Methods:**

PubMed (from 1950), the Cochrane Library, the CNKI (from 1979), the VIP (from 1989), and the Wanfang Data (from 1990) and the reference lists of the retrieved articles were searched for randomized controlled trials in human beings with the search terms including “polycystic ovary syndrome/PCOS” and “berberine/BBR/Huangliansu (in Chinese)/Xiao bojian (in Chinese)” till 30 May 2019. Relevant indicators were collected and the data were analyzed by using RevMan 5.3 software.

**Results:**

Eventually, a total of 12 randomized controlled trials were included in this systematic review. Our study suggested that berberine had similar live birth rates compared with placebo or metformin and lower live birth rates (RR: 0.61, 95% CI: 0.44 to 0.82) compared with letrozole. There was a significant difference between berberine and placebo and between berberine and no treatment in terms of decreasing total testosterone and luteinizing hormone to follicle-stimulating hormone (LH/FSH) ratio (8 RCTs, 577 participants, MD: −0.34, 95% CI: −0.47 to −0.20; 3 RCTs, 179 participants, MD: −0.44, 95% CI: −0.68 to −0.21, respectively). Berberine was associated with decreasing total cholesterol (3 RCTs, 201 participants; MD: −0.44, 95% CI: −0.60 to −0.29), waist circumference (3 RCTs, 197 participants, MD: −2.74, 95% CI: −4.55 to −0.93), and waist-to-hip ratio (4 RCTs, 258 participants, MD: −0.04, 95% CI: −0.05 to −0.03) compared with metformin, but not with improved BMI (4 RCTs, 262 participants, MD: −0.03, 95% CI: −0.46 to 0.39). Berberine did not increase the incidence of gastrointestinal adverse events (3 RCTs, 567 participants, RR: 1.01, 95% CI: 0.76 to 1.35) or serious events during pregnancy (RR: 0.98, 95% CI: 0.70 to 1.37) compared with placebo.

**Conclusion:**

This review found no solid evidence that berberine could improve live birth or other clinical outcomes in women with PCOS. However, berberine appeared to be more efficacious for improving insulin resistance and dyslipidemia and decreasing androgen levels and LH/FSH ratio in women with PCOS when compared with metformin.

## 1. Introduction

Polycystic ovary syndrome (PCOS) is a heterogeneous endocrine disorder, and the prevalence worldwide ranges from 4% to 21%, depending on different diagnostic criteria [1, 2]. Metabolic dysfunction is a key feature of PCOS, which is characterized by dysglycemia, hyperinsulinemia, and dyslipidemia. Insulin resistance has an important role in the mechanism of PCOS in both obese and nonobese women, and hyperinsulinemia in response to insulin resistance increases ovarian androgen synthesis and decreases hepatic sex-hormone binding globulin (SHBG) synthesis resulting in androgen excess [3]. Women with PCOS with oligo-ovulation or anovulation have higher homeostatic model assessment of insulin resistance (HOMA-IR) compared to those with normal menstrual cycles [4]. Insulin-sensitizing drugs, especially metformin, are widely used as second-step treatments and as cotreatments for PCOS. Some studies report that metformin might improve live birth rates compared with placebo, and coupling metformin with clomiphene citrate might improve clinical pregnancy and ovulation rates when compared with clomiphene citrate alone [5–8]. However, the evidence to support these associations is weak, and women taking metformin often suffer from gastrointestinal side effects.

Berberine, an isoquinoline derivative alkaloid isolated from Rhizoma coptidis, is commonly used to treat inflammation, diabetes mellitus, hyperlipidemia, and infertility [9–11]. Several studies indicate that berberine has similar effects as metformin on improving hyperglycemia and is more beneficial for decreasing hyperlipidemia in patients with type 2 diabetes mellitus [12–14]. Additionally, berberine has similar effects as metformin on improving metabolic index, insulin level, and hyperandrogenemia, and it has additional effects on body composition and hyperlipidemia in women with PCOS when compared with metformin [15]. Several studies indicated that berberine inhibits the mTOR pathway with abnormally high activity in the state of insulin resistance mainly by activating AMPK activity, so as to mediate the insulin signaling pathway and improve insulin resistance [16–21].

A systematic review has reported on randomized controlled trials (RCTs) comparing berberine with metformin in women with PCOS, which evaluated the effect of berberine on glucose and lipid metabolic indexes and WHR of PCOS patients [22]. However, reproductive disorders are also urgent problems for PCOS patients. Additionally, more RCTs have been published since the publication of this review [23–28]. Thus, it is necessary to assess the current trials to systematically review the potential efficacy and safety of berberine on reproductive and metabolic outcomes in women with PCOS.

The aim of this systematic review was to assess the efficacy and safety of berberine in women with PCOS in terms of reproduction outcomes, clinical symptoms, metabolic status, and hormone levels.

## 2. Materials and Methods

The review was registered with systematic review record CRD 42016044031 in the PROSPERO database.

### 2.1. Search Strategies

PubMed (from 1950), the Cochrane Library, the China National Knowledge Infrastructure (from 1979), the VIP Database for Chinese Technical Periodicals (from 1989), and the Wanfang Database on Academic Institutions in China (from 1990) were searched till 30 May 2019. Search keywords included “polycystic ovary syndrome/PCOS” and “berberine/BBR/Huangliansu (in Chinese)/Xiao bojian (in Chinese).”

### 2.2. Study Selection

To determine the studies to be searched further, two review authors (QX and DYY) independently scanned the titles and abstracts of all articles identified from electronic databases. Full-text articles were scanned for all potentially relevant articles. If there was any disagreement on the selection of articles, they discussed with the third author (XLZ).

### 2.3. Selection Criteria

According to the PRISM statement, we used the PICO (population, intervention, comparison, and outcome) framework to establish a priori selection criteria for including or excluding the studies in this systematic review.

The inclusion criterion for the population was women diagnosed with PCOS according to specific criteria, e.g., the Rotterdam criteria. The inclusion criteria for study type were RCTs. Exclusion criteria included adolescents (under 18 years of age) and postmenopausal women (over 50 years).

Interventions included berberine only (no limit to dosage form, dose, or duration) or berberine-combined interventions. Control interventions included no treatment, placebo, western medicine, herbal medicine, lifestyle intervention, and exercise. The primary outcomes were defined as live birth and adverse events. We described all adverse events reported in the included studies. Secondary outcomes included other clinical reproduction outcomes (ovulation, pregnancy, and conception), glucose and lipid metabolism (fasting and postprandial plasma glucose, fasting and postprandial insulin, total triglycerides, total cholesterol, high-density lipoprotein (HDL), and low-density lipoprotein (LDL)), clinical symptoms (body mass index (BMI), waist circumference (WC), and waist-to-hip ratio (WHR)), and reproductive hormones (total testosterone, free testosterone, free androgen index (FAI), luteinizing hormone (LH), follicle-stimulating hormone (FSH), and LH-to-FSH ratio).

### 2.4. Risk of Bias Appraisal

The methodological quality of the included trials was assessed independently by two authors (YYD and QX) using the Cochrane risk of bias tool (the Cochrane Handbook for Systematic Reviews of Interventions [29]). Individual quality items were investigated using a descriptive component approach that included the six special domains of sequence generation, allocation concealment, blinding, incomplete outcome data, selective outcome reporting, and other bias. The six domains were categorized as “yes,” “no,” and “unclear,” and all disagreements were resolved through discussion (with XKW and LZX) to reach a consensus.

### 2.5. Data Extraction

Data were extracted from the included studies by two independent reviewers (YYD and QX) using a specially developed data extraction form according to the selection criteria. Information extracted included descriptions of the studies (authors, country, year of publication, diagnostic criteria for PCOS, primary and secondary outcomes, sample size, and follow-up), participants (mean age and BMI), interventions (type, dose, and duration of berberine), and study results according to the outcomes outlined above (Table 1).

### 2.6. Strategy for Data Synthesis

Review Manager software version 5.3 by the Cochrane Collaboration Network was used for data analysis. Continuous data were expressed as the mean difference (MD) with 95% confidence interval (CI), and dichotomous data were presented as risk ratio (RR) with 95% CI. Meta-analyses were performed with the included RCTs, and the heterogeneity was evaluated with the Higgins *I*^2^ test. If *I*^2^ > 50%, a random effects model was used for meta-analyses of the data. If not, a fixed effects model was used. Sensitivity analysis was carried out to explore heterogeneity due to extreme data. The funnel plot was used to detect small-study effects or publication biases.

## 3. Results

### 3.1. Study Selection

**Figure 1:**
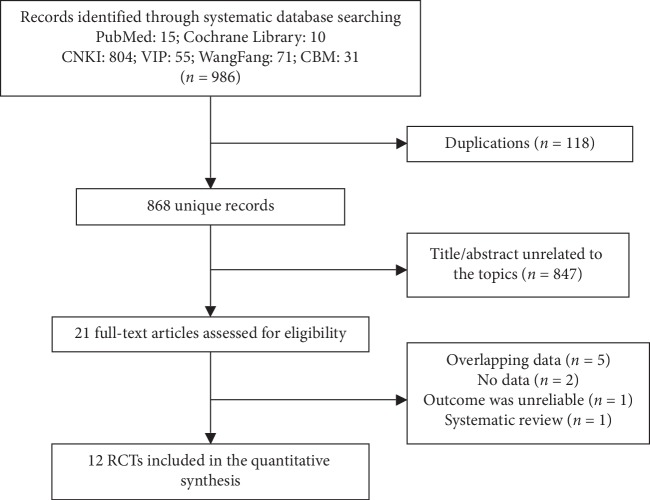
Flow chart.

Of the 986 articles identified in the initial searches, 21 were selected for full review, including 12 RCTs [15, 23–28, 30–34] (Figure 1). The 12 RCTs were included in the analysis and comprised a total of 1,544 women with PCOS based on eligibility criteria. The 12 RCTs had samples ranging from 50 to 644 women with PCOS, the majority of whom were under 36 years of age. Ten of the studies reported diagnostic criteria included in the 2003 Rotterdam ESHRE/ASRM criteria [35]. Berberine alone or combined with drugs or assisted reproductive technology were used as the interventions, and the controls were placebo or no intervention. In most trials, all women generally received 900 mg or 1,500 mg berberine per day, except for three trials in which the berberine intake was 2,000 mg per day. The duration of berberine treatment ranged from 3 to 24 weeks.

### 3.2. Risk of Bias

The risks of bias are summarized in Supplementary [Supplementary-material supplementary-material-1]. Eight studies reported sequence generation, and four of these only reported the details of allocation concealment. One study reported double-blinding and one reported single-blinding. All studies but one reported the reasons for withdrawals if there were withdrawals. One study had a high risk of selective reporting bias.

### 3.3. Berberine vs. Placebo or No Treatment

#### 3.3.1. Primary Outcome

**Figure 2:**
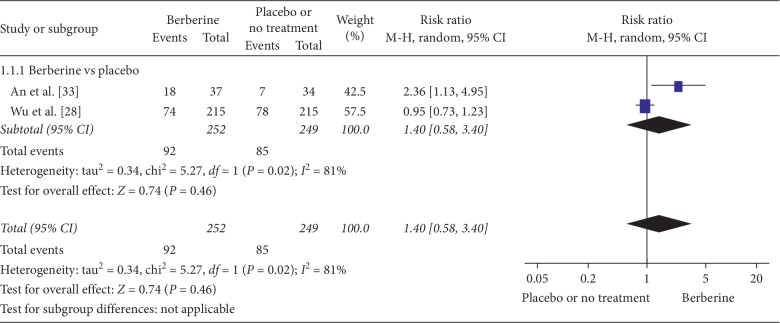
Meta-analyses of the effect of berberine on fertility outcome (Live birth) compared with placebo or no treatment.

Four studies [26, 28, 30, 33] compared berberine with placebo, and two of these reported live birth rates [28, 33]. Combined with lifestyle, berberine was associated with a higher live birth rate compared with placebo (RR 2.36, 95% CI 1.13 to 4.95) prior to IVF/ICS treatment (Figure 2) [33]. However, in combination with letrozole, the incidence of live birth was similar for berberine vs. placebo (RR 0.95, 95% CI 0.73 to 1.23) (Figure 2) [28]. No studies reported that berberine improved live birth when it was used alone.

#### 3.3.2. Other Clinical Outcomes

All four RCTs reported clinical reproductive outcomes [26, 28, 33, 34]. There was no evidence that berberine was associated with higher pregnancy compared with placebo or no treatment (4 RCTs, 620 participants, RR: 1.46, 95% CI: 0.90 to 2.36, *I*^2^ = 62%) (Table 2) [26, 28, 33, 34]. However, subgroup analysis showed that berberine combined with other treatments improved pregnancy (2 RCTs, 119 participants; RR: 2.05, 95% CI: 1.16 to 3.64, *I*^2^ = 0) compared with the other treatment alone [26, 28]. Berberine was associated with higher ovulation per cycle, but similar ovulation per subject (RR: 1.51, 95% CI: 1.23 to 1.86; RR: 1.21, 95% CI: 0.95 to 1.54, respectively), in women with PCOS compared with no treatment (Table 2) [26].

#### 3.3.3. Reproductive Hormone Levels

Eight studies assessed total testosterone comparing berberine with placebo or no treatment [15, 26, 27, 30–34]. A meta-analysis was performed and showed that total testosterone was slightly but significantly decreased in the berberine group compared with placebo or no treatment (8 RCTs, 577 participants; MD: −0.34, 95% CI: −0.47 to −0.20, *I*^2^ = 80%) (Table 2) [15, 26, 27, 30–34]. Sensitivity analysis by study quality showed that there was a barely detectable but statistically significant difference between berberine and placebo or no treatment, but no improvement in the heterogeneity, in two of the included studies [15, 33]. Berberine treatment compared with placebo yielded slightly increased SHBG (2 RCTs, 146 participants, MD: 13.71, 95% CI: 8.93 to 18.48, *I*^2^ = 0) [15, 33] and decreased FAI (2 RCTs, 146 participants; MD: −1.30, 95% CI: –1.73 to −0.88, *I*^2^ = 0) (Table 2) [15, 33].

Berberine was associated with decreased LH compared with placebo (3 RCTs, 196 participants, MD: −1.04, 95% CI: −1.87 to −0.21, *I*^2^ = 47%) (Table 2) [15, 30, 33]. There was a significant difference between berberine and no treatment in term of decreasing LH (5 RCTs, 381 participants, MD: −1.49, 95% CI: −2.26 to −0.73, *I*^2^ = 67%) [26, 27, 31, 32, 34] and decreasing LH-to-FSH ratio (3 RCTs, 179 participants, MD: −0.44, 95% CI: −0.68 to −0.21, *I*^2^ = 53%) (Table 2) [27, 32, 34].

#### 3.3.4. Metabolic Characteristics

Seven studies reported metabolic characteristics, including glucose and lipid profiles [15, 23, 26, 30, 31, 33, 34]. For the glucose profile, berberine was associated with decreasing fasting plasma glucose (3 RCTs, 196 participants, MD: −0.35, 95% CI: −0.55 to −0.16, *I*^2^ = 41%) [15, 30, 33] and decreasing insulin levels (2RCTs, 146 participants, MD: −5.86, 95% CI: −7.99 to −3.74, *I*^2^ = 64%) [15, 33] compared with placebo (Table 2). Berberine had a decreasing postprandial plasma glucose level (MD: −0.60, 95% CI: −0.98 to −0.22) [15] compared with placebo (Table 2) and lower HOMA-IR (MD: −2.20, 95% CI: −2.68 to −1.72) [34] compared with no treatment (Table 2).

For the lipid profiles, berberine was associated with decreasing total cholesterol (6 RCTs, 457 participants, MD: −0.53, 95% CI: −0.68 to −0.38, *I*^2^ = 65%) [15, 23, 26, 30, 31, 33], triglycerides (6 RCTs, 457 participants, MD: −0.18, 95% CI: −0.25 to −0.12, *I*^2^ = 58%) [15, 23, 30, 31, 33, 34], and LDL-C (4 RCTs, 320 participants, MD: −0.34, 95% CI: −0.42 to −0.26, *I*^2^ = 45%) [15, 23, 31, 34] and increasing HDL-C (4 RCTs, 311 participants, MD: 0.12, 95% CI: 0.09 to 0.14, *I*^2^ = 0) [15, 23, 31, 34] compared with placebo or no treatment (Table 2). Sensitivity analysis by study quality did not change the inference in total cholesterol and did not improve heterogeneity but decreased heterogeneity in triglycerides in two studies [15, 33].

#### 3.3.5. Obesity

Seven studies addressed obesity indexes, including BMI, WC, and WHR [15, 27, 30–34]. BMI was slightly increased, but not significantly, for berberine compared to placebo or no treatment (7 RCTs, 497 participants, MD: −0.67, 95% CI: −1.38 to 0.04, *I*^2^ = 81%) [15, 27, 30–34] (Table 2). Sensitivity analysis by study quality did not change the inference and did not improve heterogeneity in two of the studies [15, 33]. Compared with placebo, one study indicated that berberine reduced WC (MD: −3.40, 95% CI: −5.63 to −1.17) [33] prior to IVF/ICS treatment. However, in another study, there was no significant difference between berberine and placebo in terms of reducing WC (MD: −0.53, 95% CI: −3.20 to −2.14) [15]. Additionally, a meta-analysis showed that WHR was slightly but significantly decreased in the berberine groups versus placebo or no treatment (5 RCTs, 402 participants, MD: −0.04, 95% CI: −0.05 to −0.03, *I*^2^ = 0) (Table 2) [15, 27, 30, 31, 33].

#### 3.3.6. Adverse Events

Three studies addressed adverse events in the berberine group versus placebo [15, 30, 33]. Berberine was associated with similar gastrointestinal adverse events (3 RCTs, 567 participants, RR: 1.01, 95% CI: 0.76 to 1.35, *I*^2^ = 0) [28, 30, 33] compared with placebo (Table 2). There was no difference in serious events during pregnancy between berberine and placebo (RR: 0.98, 95% CI: 0.70 to 1.37) [28] (Table 2).

### 3.4. Berberine vs. Metformin

#### 3.4.1. Primary Outcome

One study reported live birth rates for berberine vs. metformin [33]. This study showed that the incidence of live birth was slightly higher but not significant in women with PCOS treated with berberine compared with metformin (RR: 1.32, 95% CI: 0.78 to 2.25) [33], although the original article indicated that there were differences between berberine and metformin (Table 2).

#### 3.4.2. Other Clinical Outcomes

**Figure 3:**
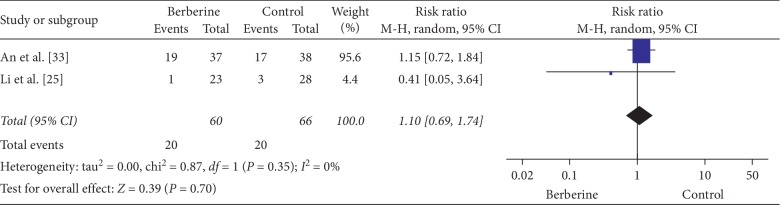
Meta-analyses of the effect of berberine on pregnancy compared with metformin.

Two studies [25, 33] reported pregnancy per subject, and one [24] reported ovulation per subject. Berberine had similar pregnancy per subject (2 RCTs, 126 participants, RR: 1.10, 95% CI: 0.69 to 1.74, *I*^2^ = 0) [25, 33] compared with metformin (Figure 3) but higher ovulation per subject (RR: 1.32, 95% CI: 1.03 to 1.70) (Table 2) [24].

In combination with IVF/ICS and lifestyle intervention, berberine was associated with similar pregnancy rates compared with metformin (RR: 1.15, 95% CI: 0.72 to 1.84) [33], while berberine alone was associated with similar pregnancy rates compared with metformin (RR: 0.41, 95% CI: 0.05 to 3.64) [25] (Figure 3).

#### 3.4.3. Reproductive Hormone Levels

**Figure 4:**
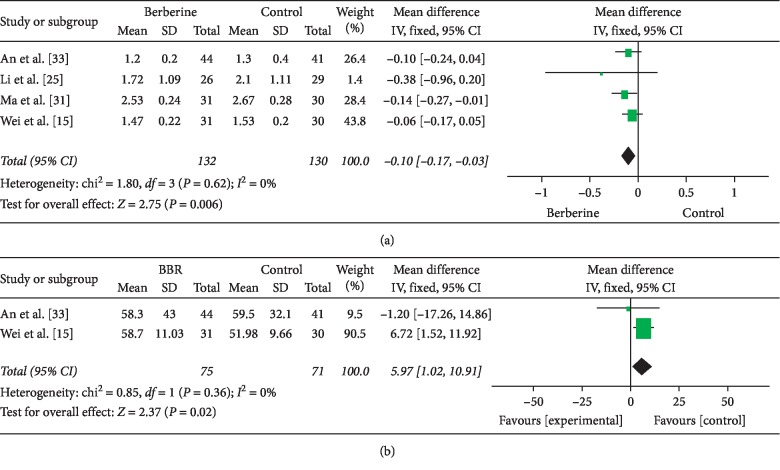
Meta-analyses of the effect of berberine on reproductive endocrinology indexes: (a) total testosterone and (b) sex-hormone binding globulin compared with metformin.

Four studies assessed total testosterone comparing berberine with metformin. A meta-analysis showed that total testosterone was slightly but significantly decreased in the berberine versus metformin groups (4 RCTs, 262 participants, MD: −0.10, 95% CI: −0.17 to −0.03, *I*^2^ = 0) [15, 25, 31, 33] (Figure 4(a)). There was a significant difference in SHBG between berberine and metformin (2 RCTs, 146 participants, MD: 5.97, 95% CI: 1.02 to 10.91) [15, 33] (Figure 4(b)). Berberine had slightly decreased LH and FSH but did not quite achieve significance (4 RCTs, 262 participants, MD: −0.49, 95% CI: −1.31 to 0.33, *I*^2^ = 0; MD: −0.17, 95% CI: −0.79 to 0.45, *I*^2^ = 82%, respectively) [15, 25, 31, 33] (Table 2). However, there was significant decrease in LH/FSH ratio between berberine and metformin (MD: −0.90, 95% CI: −1.58 to −0.22) [25] (Table 2).

#### 3.4.4. Metabolic Characteristics

For the glucose profile, four studies addressed glucose and insulin levels in the berberine group versus controls [15, 25, 31, 33]. There was no significant difference between berberine and metformin in terms of reducing fasting plasma glucose, postprandial plasma glucose, fasting insulin, or HOMA-IR (4 RCTs [15, 25, 31, 33], 262 participants, MD: −0.03, 95% CI: −0.23 to 0.16, *I*^2^ = 52%; 2 RCTs [15, 25], 116 participants, MD: −0.13, 95% CI: −0.51 to 0.25, *I*^2^ = 0; 4 RCT [15, 25, 31, 33], 262 participants; MD: −0.95, 95% CI: −2.09 to 0.20, *I*^2^ = 12%; 4 RCT [15, 25, 31, 33], 262 participants; MD: −0.22, 95% CI: −0.47 to 0.02, *I*^2^ = 0%, respectively) (Table 2).

**Figure 5:**
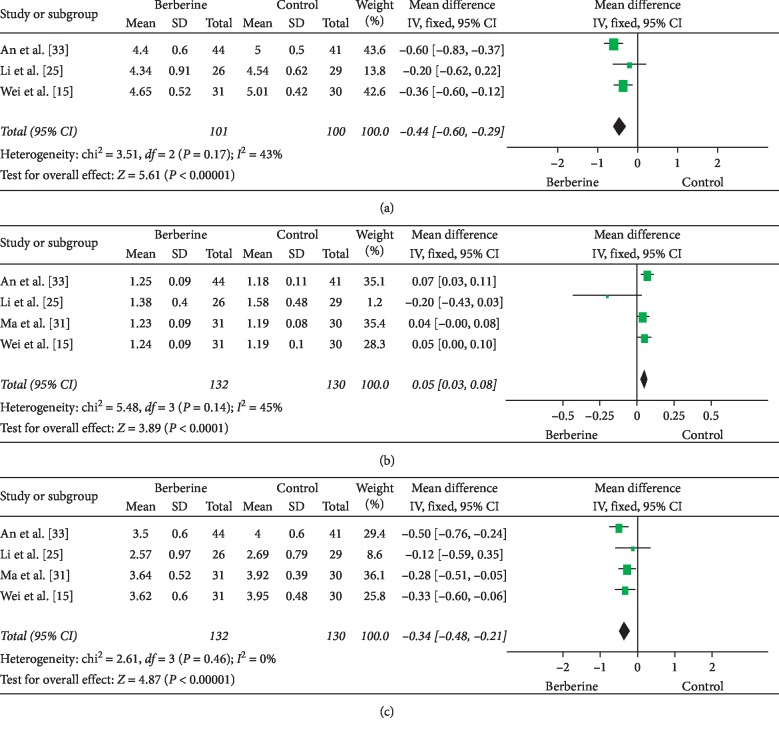
Meta-analyses of the effect of berberine on metabolic characteristics: (a) total cholesterol; (b) HDL-C; and (c) LDL-C compared with metformin.

For the lipid profiles, four studies addressed various aspects of lipid metabolism for berberine versus metformin [15, 25, 31, 33]. Berberine had slightly lower total cholesterol (3 RCTs, 201 participants, MD: −0.44, 95% CI: −0.60 to −0.29, *I*^2^ = 43%) [15, 25, 33] and LDL levels (4 RCTs, 262 participants, MD: −0.34, 95% CI: −0.48 to −0.21, *I*^2^ = 0) [15, 25, 31, 33] and slightly higher HDL levels (4 RCTs, 262 participants, MD: 0.05, 95% CI: 0.03 to 0.08, *I*^2^ = 45%) [15, 25, 31, 33] compared with metformin (Figures 5(a)–5(c)).

#### 3.4.5. Obesity

**Figure 6:**
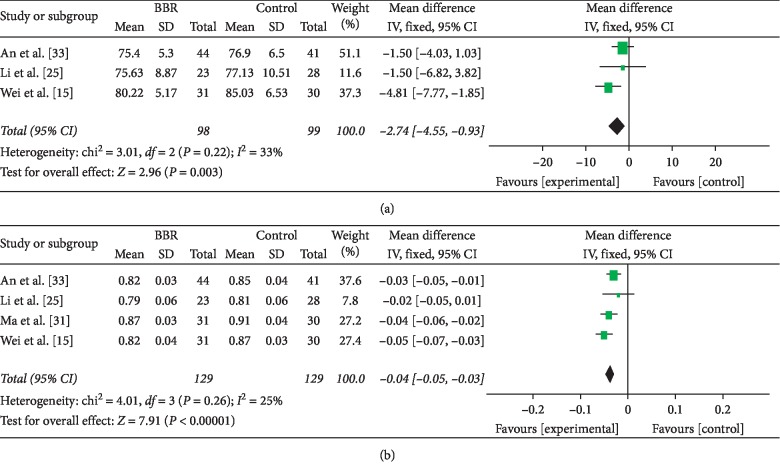
Meta-analyses of the effect of berberine on adiposis: (a) waist circumference and (b) waist-to-hip ratio compared with metformin.

Four studies [15, 25, 31, 33] addressed obesity indexes, including BMI, WC (except for Ma 2011 [31]), and WHR. Berberine was associated with slightly decreased BMI, but not significantly, compared with metformin (4 RCTs, 262 participants, MD: −0.03, 95% CI: −0.46 to 0.39, *I*^2^ = 31%) [15, 25, 31, 33] (Table 2). WC (3 RCTs, 197 participants, MD: −2.74, 95% CI: −4.55 to −0.93, *I*^2^ = 33%) [15, 25, 33] and WHR (4 RCTs, 258 participants, MD: −0.04, 95% CI: −0.05 to −0.03, *I*^2^ = 25%) [15, 25, 31, 33] were slightly but significantly lower in the berberine group (Figures 6(a) and 6(b)).

#### 3.4.6. Adverse Events

**Figure 7:**
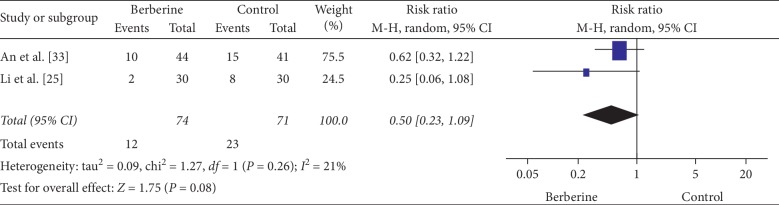
Meta-analyses of the effect of berberine on gastrointestinal adverse events compared with metformin.

Two studies addressed adverse events [25, 33]. Berberine was associated with a similar incidence of gastrointestinal adverse events compared with metformin prior to IVF/ICS intervention (RR: 0.62, 95% CI: 0.32 to 1.22) [33] (Figure 7) and compared with metformin alone (RR: 0.25, 95% CI: 0.06 to 1.08) [25] (Figure 7).

### 3.5. Berberine vs. Letrozole

#### 3.5.1. Primary Outcome

One study [28] reported live birth rate in berberine vs. letrozole treatments. The study showed that berberine was associated with a lower live birth rate (RR: 0.61, 95% CI: 0.44 to 0.82) [28] compared with letrozole (Table 2).

#### 3.5.2. Other Clinical Outcomes

There was only one study that reported other reproductive outcomes in berberine vs. letrozole treatments [28]. Berberine had significantly lower pregnancy, conception, ovulation per subject, and ovulation per cycle (RR: 0.57, 95% CI: 0.43 to 0.77; RR: 0.63, 95% CI: 0.48 to 0.81; RR: 0.79, 95% CI: 0.71 to 0.87; and RR: 0.61, 95% CI: 0.55 to 0.68, respectively) [28] compared with letrozole (Table 2).

#### 3.5.3. Adverse Events

One study reported adverse events in the berberine vs. letrozole treatments [28]. Gastrointestinal adverse events were slightly higher for women with berberine (RR: 1.32, 95% CI: 1.00 to 1.73) [28] when compared to those with letrozole (Table 2). Serious events during pregnancy were slightly lower but not significant for women with berberine (RR: 0.87, 95% CI: 0.61 to 1.24) [28] (Table 2).

## 4. Discussion

This study was a comprehensive systematic review to evaluate the effect of berberine in women with PCOS. In this study, we not only evaluated the lipid-lowering and glucose-lowering properties of berberine in PCOS patients, as seen in studies on cardiovascular disease, but also evaluated the efficacy of berberine in reproductive hormone production and reproductive outcomes.

**Figure 8:**
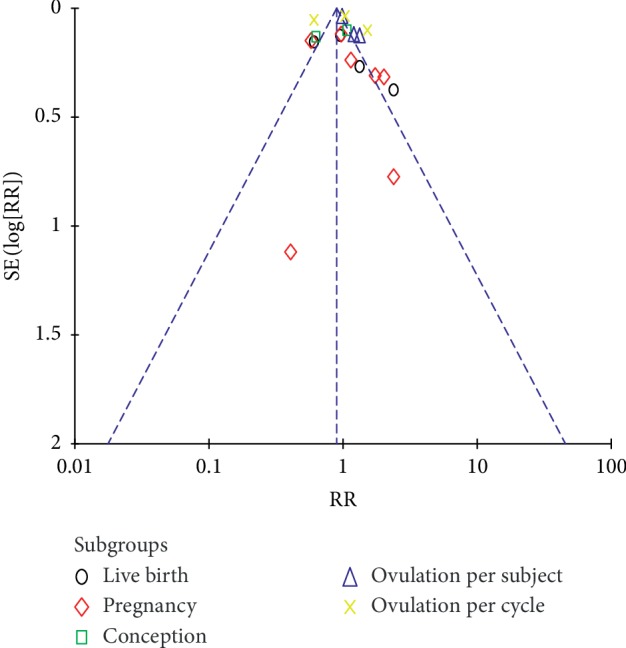
Funnel plot for published studies of cslinical reproduction outcomes.

Our analysis of berberine for improving fertility in PCOS patients showed similar effectiveness as letrozole with no significant increase in the live birth rate or ovulation rate. However, the use of berberine alone achieved a 36% ovulation rate per cycle, similar to metformin, and a 22% cumulative live birth rate, similar to clomiphene, after 6 months of use [36]. The biochemical and clinical pregnancy rates and live birth rate were significantly higher in the berberine groups compared with placebo prior to IVF/ICS treatment. The total FSH dosages used for ovarian stimulation were significantly lower in the berberine group than in the metformin and placebo groups. Moreover, both berberine and metformin reduced the incidence of severe ovarian hyperstimulation syndrome [24]. The funnel plot for reproductive outcomes indicated that there were no small-study effects or publication bias (Figure 8). Taken together, these results suggest that berberine improves fertility in women with PCOS.

There is now an extensive body of evidence demonstrating that insulin can increase circulating androgen levels in women with PCOS [37, 38] and that theca cells from women with PCOS are more responsive to the androgen-stimulating actions of insulin than those from control women [39]. Under physiological circumstances, insulin most likely acts as a co-gonadotropin to increase LH-induced androgen synthesis in theca cells [40–42]. In theca cells, insulin works synergistically with LH to activate the 17-hydroxylase activity of P450c17, a key enzyme in the regulation of androgen biosynthesis encoded by the *CYP17* gene, via PI3-K signaling, and inhibition of MAPK-ERK1/2 signaling has no effect on 17-hydroxylase activity [40]. In addition, increased insulin levels in synergy with LH in granulosa cells from anovulatory polycystic ovaries might trigger premature LH receptor expression in a subpopulation of small follicles leading to premature granulosa terminal differentiation and the arrest of follicular growth that might contribute to anovulation in this subgroup [43, 44].

Ovarian granulosa cells from porcine follicles were isolated and cultured in vitro to establish an insulin resistance model induced by dexamethasone, and these cells had significantly lower [3H]-glucose uptake and significantly higher testosterone levels. After berberine treatment, the mRNA and protein analyses of these cells showed elevated expression of IGF-1R, IRS-1, PI-3K, Akt2, and GLUT4 but reduced expression of PPAR-*γ* and aromatase, suggesting an improvement in both insulin sensitivity and steroidogenesis in granulosa cells [45, 46]. The findings of this study confirmed that berberine can significantly reduce total testosterone and FAI and increase SHBG compared with placebo or no treatment and that there are significant differences between berberine and metformin in terms of decreasing total testosterone and increasing SHBG.

Insulin can also enhance GnRH-mediated LH and FSH release from cultured rat pituitary cells [47]. Furthermore, female mice with hyperinsulinemia secondary to diet-induced obesity have increased basal and GnRH-stimulated LH release [48]. In our study, berberine was associated with lower LH compared with placebo and with lower LH and LH/FSH compared with no treatment. There were significant differences between berberine and metformin in terms of decreasing LH/FSH.

Previous studies have shown that berberine shows good potential for the prevention and treatment of metabolic disorders, including cholesterol reduction and antilipogenic and hypoglycemic effects [49–53]. In our study, berberine was associated with lower fasting glucose compared with placebo and with significantly reduced 2-hour glucose compared to no treatment. There were no differences between berberine and metformin in terms of decreasing fasting glucose, 2-hour glucose, fasting insulin, 2-hour insulin, or HOMA-IR. These results are consistent with the previous systematic review on evaluating the effect of berberine on PCOS with IR [54]. A growing body of evidence suggests that berberine improves insulin sensitivity and stimulates glucose uptake via activation of the AMP-activated protein kinase pathway [16–19], inhibition of gluconeogenesis [55], promotion of glycolysis [56], and increasing glucose transporter expression [57], and thus, berberine promotes glucose transport and enhances glucose metabolism.

D-chiro-inositol (a polyalcohol classified as a secondary messenger in insulin signaling) is commonly applied as insulin sensitizers to increase insulin sensitivity of PCOS. Several studies showed that the combination of d-chiro-inositol (DCI) and alpha-lipoic acid can improve the insulin resistance and menstrual cycle of PCOS patients [58, 59], while there was no statistically difference in total cholesterol and triglycerides levels when compared with the control group [59]. Our research showed that there were significant differences in terms of decreasing total cholesterol, triglycerides, and LDL-C and increasing HDL-C, between berberine and placebo, no treatment, and metformin. Berberine can increase the oxidation of free fatty acids [60], upregulate the expression of LDL receptor in hepatocytes [52, 61] through activation of extracellular regulated protein kinases, and inhibit the synthesis of glycerol three lipid and cholesterol in the liver [62] through activation of AMP kinase, which improves hepatocyte insulin resistance and lipid metabolism.

Our research showed that berberine had similar BMI and WC, but lower WHR, compared with placebo and with no treatment. There were no differences between berberine and metformin in terms of decreasing BMI, but significant differences in terms of decreasing WC and WHR. These results provide supporting evidence for berberine-induced adipose tissue redistribution and amelioration of central fat distribution, which might consequently affect insulin sensitivity independent of changes in body weight.

We also found that berberine had a similar incidence of gastrointestinal adverse events and serious adverse events during pregnancy compared to placebo, metformin, and letrozole, which was due to the limited number of included RCTs included in this analysis. The major side effects of berberine can result from overdose, including diarrhea, constipation, flatulence, and abdominal pain in rare cases [52]. A detailed study of berberine showed no elevation in biochemical parameters, including transaminases (AST and ALT), g-GT, and CPK, thus demonstrating the safety of berberine [63], and the pharmacokinetics of berberine in rats suggests that blood clearance of berberine is very quick and that its biotransformation in the liver is rapid [64].

There was a systematic review and meta-analysis on evaluating the effect of berberine on PCOS with IR published previously [54]. A total of 9 RCTs were included in this systematic review. There were 8 RCTs overlapped with our study. The former review focused on evaluating the synergistic effects of berberine combined with metformin or contraceptives. Our study found that berberine and metformin have similar effect on reducing IR, and berberine is superior to metformin in reducing total testosterone level and improving blood lipid and body fat distribution.

Nevertheless, this study had several important limitations that are common to this type of study. First, all included trials were conducted among Chinese women with PCOS in mainland China. Due to a high risk of selection bias, we are not sure whether we would expect to find similar results in other ethnicities or races. Second, most of the studies were of low methodological quality, although most addressed the method of randomization sequence generation. Four studies performed adequate allocation concealment, but only two used blinding. Additionally, one study was likely to have attrition bias and one to have selective bias. Therefore, potential bias in selection of participants and treatment and assessment of outcomes might result in overrating the efficacy of berberine. Third, the heterogeneity between the included trials was significant. However, because of the lack of original research data on individual participants, we could not perform subgroup analyses or regression analyses. Thus the results are limited and it is difficult to draw solid conclusions about the efficacy of berberine in treating PCOS.

## 5. Conclusion

Our review of available RCTs suggests that berberine might be useful in restoring normal endocrinological and fertility. In women with PCOS, and compared with metformin, berberine can significantly reduce total testosterone, plasma lipid, WC, and WHR and increase SHBG. Berberine has a low documentation of adverse effects in humans, and thus, berberine appears to be a useful and safe drug for improving spontaneous ovulation and enhancing fertility.

## Figures and Tables

**Table 1:** Characteristics of randomized controlled trials included in this systematic review and meta-analyses.

Study	Study location	Sample size	Age (year) (mean ± SD)	Diagnostic criteria	Treatment group			Outcomes
					Treatments	Dosage	Duration (weeks)	
An et al. [33]	China	50	28.2 ± 3.8	2003 Rotterdam	BBR	500 mg, tid	12	Live birth pregnancy, adverse events, BMI, WC, WHR
		50	28.7 ± 4.2		MET	500 mg, tid		SHBG, FAI, TT, LH, FSH, FPG, FINS, TC, TG, HOMA, HDL, LDL
		50	28.4 ± 4.0		Placebo	1 pill, tid		

Chen et al. [23]	China	50	25.9 ± 2.5	UK	CPA	1 pill, qd	3	TC, TGHDL, LDL
		50	26.1 ± 2.5		BBR + CPA	1000 mg/d, bid		

Li and Yu [24]	China	53	18–36	2003 Rotterdam	BBR + LET	500 mg, tid	12	Ovulation (subject)
		45			MET + LET	500 mg, tid		

Li et al. [25]	China	30	24.61 ± 4.79	2003 Rotterdam	BBR	300 mg, tid	12	Pregnancy, TT, BMI, WC, WHR, FPG, PPG, FINS, PPINS, LH, FSH, LF, HOMA, TC, TG, HDL, LDL
		30	26.86 ± 4.04		MET	500 mg, tid		

Liu [34]	China	23	26.09 ± 4.63	2003 Rotterdam	Herbal	400 mg, tid	12	Pregnancy, BMI, LH, FSH, LF, TT, FPG, FINS, HOMA, TG, LDL, HDL
		23	25.70 ± 4.68		Herbal + BBR			

Liu et al. [25]	China	40	26.52 ± 6.85	2003 Rotterdam	BBR + CC + CPA	1000 mg, tid	12	Pregnancy, ovulation (cycle), FPG, FINS, TC, SHBG, TT, LH
		40	27.53 ± 8.16		CC + CPA			

Ma et al. [31]	China	28	26.75 ± 2.62	2003 Rotterdam	CPA	1 pill, qd	12	FPG, FINS, BMI, TC, WHR, TG, TT, LH, FSH, HDL, LDL, HOMA
		31	25.74 ± 2.66		CPA + BBR	1000 mg, bid		
		30	26.03 ± 2.82		CPA + MET	500 mg, tid		
		33	26.27 ± 1.89		CPA + BBR + MET			

Wang et al. [32]	China	28	25.5 ± 3.2	2003 Rotterdam	MET	500 mg, tid	12	BMI, TT, LH, FSH, LF
		28			MET + BBR	500 mg, tid		

Wang et al. [27]	China	42	24.5 ± 3.4	UK	MET	500 mg, tid	12	Ovulation (subject), BMI, WHR, TT, LH, FSH, LF
		42	24.3 ± 3.5		MET + BBR	500 mg, tid		

Wei et al. [15]	China	31	26.75 ± 2.62	2003 Rotterdam	Placebo + CPA	1 pill, qd	12	BMI, WC, WHR, FPG, FINS, PPG, TC, TG, LDL, HDL
		35	25.74 ± 2.66		BBR + CPA	500 mg, tid		SHBG, TT, FAI, LH, FSH, HOMA
		34	26.03 ± 2.82		MET + CPA	500 mg, tid		

Wu et al. [28]	China	214	27.8 ± 3.7	2003 Rotterdam	BBR + Placebo	1500 mg, qd	24	Live birth pregnancy, conception, ovulation (subject cycle), adverse events
		215	27.8 ± 3.6		LET + Placebo	2.5 mg, qd		
		215	27.8 ± 3.6		BBR + LET			

Zhu et al. [30]	China	25	47.64 ± 8.32	2003 Rotterdam	Placebo + CPA	1 pill, qd	3	BMI, WHR, FPG, TC, TG, TT, adverse events, LH, FSH
		25	48.36 ± 8.45		BBR + CPA	300 mg, tid		

2003 Rotterdam: 2003 Rotterdam ESHRE/ASRM criteria; BBR: berberine; MET: metformin; CPA: cyproterone; LET: letrozole; BMI: body mass index; WC: waist circumference; WHR: waist circumference-to-hip circumference ratio; TT: total testosterone; SHBG: sex-hormone binding globulin; FAI: free androgen index; LH: luteinizing hormone; FSH: follicle-stimulating hormone; LF: luteinizing hormone to follicle-stimulating hormone ratio; FPG: fasting plasma glucose; PPG: postprandial plasma glucose; FINS: fasting insulin; PPINS: postprandial plasma insulin; HOMA: homeostasis model assessment of insulin resistance; TC: total cholesterol; TG: triglycerides; HDL: high-density lipoprotein cholesterol; LDL: low-density lipoprotein cholesterol.

**Table 2:** Data and analyses of RCTs included in this systematic review and meta-analysis.

Outcome or Subgroup	Studies	Participants	Risk ratio/mean difference 95% CI	*Z*-value	*P* value	*I* ^2^ (%)
*Berberine vs. placebo or no treatment*						
Live birth	2	501	1.40 [0.58, 3.40]	0.74	0.46	81
(1) Berberine vs. placebo	2	501	1.40 [0.58, 3.40]	0.74	0.46	81
Pregnancy	4	620	1.46 [0.90, 2.36]	1.55	0.12	62
(1) Berberine vs. placebo	2	501	1.21 [0.69, 2.14]	0.66	0.51	68
(2) Berberine vs. no treatment	2	119	2.05 [1.16, 3.64]	2.45	0.01	0
Conception	1	430	1.07 [0.88, 1.31]	0.68	0.50	—
(1) Berberine vs. placebo	1	430	1.07 [0.88, 1.31]	0.68	0.50	—
Ovulation per cycle	2	1,797	1.23 [0.84, 1.80]	1.07	0.28	92
(1) Berberine vs. placebo	1	1,593	1.03 [0.95, 1.11]	0.63	0.53	—
(2) Berberine vs. no treatment	1	204	1.51 [1.23, 1.86]	3.98	<0.001	—
Ovulation per subject	2	514	1.05 [0.86, 1.29]	0.50	0.61	63
(1) Berberine vs. placebo	1	430	0.98 [0.91, 1.05]	0.56	0.57	—
(2) Berberine vs. no treatment	1	84	1.21 [0.95, 1.54]	1.51	0.13	—
TT	8	577	−0.34 [−0.47, −0.20]	4.94	<0.001	80
(1) Berberine vs. placebo	3	196	−0.25 [−0.48, −0.02]	2.14	0.03	82
(2) Berberine vs. no treatment	5	381	−0.38 [−0.55, −0.21]	4.49	<0.001	76
SHBG	3	226	8.95 [−0.60, 18.50]	1.84	0.07	86
(1) Berberine vs. placebo	2	146	13.71 [8.93, 18.48]	5.63	<0.001	0
(2) Berberine vs. no treatment	1	80	2.41 [−0.97, 5.79]	1.40	0.16	—
FAI	2	146	−1.30 [−1.73, −0.88]	5.99	<0.001	0
(1) Berberine vs. placebo	2	146	−1.30 [−1.73, −0.88]	5.99	<0.001	0
LH	8	577	−1.29 [−1.84, −0.75]	4.66	<0.001	595
(1) Berberine vs. placebo	3	196	−1.04 [−1.87, −0.21]	2.45	0.01	47
(2) Berberine vs. no treatment	5	381	−1.49 [−2.26, −0.73]	3.83	<0.001	67
FSH	7	497	−0.21 [−1.77, 1.36]	0.26	0.80	99
(1) Berberine vs. placebo	3	196	0.17 [−0.19, 0.53]	0.94	0.35	0
(2) Berberine vs. no treatment	4	301	−0.40 [−2.78, 1.98]	0.33	0.74	99
LF	3	179	−0.44 [−0.68, −0.21]	3.65	<0.001	53
(1) Berberine vs. no treatment	3	179	−0.44 [−0.68, −0.21]	3.65	<0.001	53
FPG	5	315	−0.33 [−0.49, −0.17]	3.94	<0.001	0
(1) Berberine vs. placebo	3	196	−0.35 [−0.55, −0.16]	3.50	<0.001	41
(2) Berberine vs. no treatment	2	119	−0.28 [−0.57, 0.02]	1.84	0.07	0
PPG	1	59	−0.60 [−0.98, −0.22]	3.08	<0.001	—
(1) Berberine vs. placebo	1	59	−0.60 [−0.98, −0.22]	3.08	<0.001	—
FINS	4	265	−4.33 [−7.11, −1.55]	3.05	<0.001	92
(1) Berberine vs. placebo	2	146	−5.86 [−7.99, −3.74]	5.41	<0.001	64
(2) Berberine vs. no treatment	2	119	−2.79 [−5.72, 0.15]	1.86	0.06	84
HOMA	1	39	−2.20 [−2.68, −1.72]	8.92	<0.001	—
(1) Berberine vs. no treatment	1	39	−2.20 [−2.68, −1.72]	8.92	<0.001	—
TC	6	457	−0.53 [−0.68, −0.38]	7.11	<0.001	65
(1) Berberine vs. placebo	3	196	−0.60 [−0.84, −0.37]	5.06	<0.001	62
(2) Berberine vs. no treatment	3	261	−0.48 [−0.68, −0.28]	4.62	<0.001	72
TG	6	457	−0.18 [−0.25, −0.12]	5.54	<0.001	58
(1) Berberine vs. placebo	3	196	−0.20 [−0.37, −0.04]	2.49	0.01	84
(2) Berberine vs. no treatment	3	261	−0.18 [−0.23, −0.12]	6.28	<0.001	0
HDL	4	311	0.12 [0.09, 0.14]	9.67	<0.001	0
(1) Berberine vs. placebo	1	59	0.11 [0.06, 0.16]	4.18	<0.001	—
(2) Berberine vs. no treatment	3	252	0.12 [0.09, 0.14]	8.72	<0.001	0
LDL	4	320	−0.34 [−0.42, −0.26]	8.07	<0.001	45
(1) Berberine vs. placebo	3	196	−0.10 [−0.93, 0.72]	2.97	<0.001	100
(2) Berberine vs. no treatment	4	301	−1.06 [−2.13, 0.01]	7.53	<0.001	56
BMI	7	497	−0.67 [−1.38, 0.04]	1.86	0.06	81
(1) Berberine vs. placebo	3	196	−0.10 [−0.93, 0.72]	0.25	0.80	67
(2) Berberine vs. no treatment	4	301	−1.06 [−2.13, 0.01]	1.94	0.05	85
WC	2	146	−2.06 [−4.87, 0.74]	1.44	0.15	62
(1) Berberine vs. placebo	2	146	−2.06 [−4.87, 0.74]	1.44	0.15	62
WHR	5	402	−0.04 [−0.05, −0.03]	9.43	<0.001	0
(1) Berberine vs. placebo	3	196	−0.04 [−0.06, −0.03]	6.72	<0.001	0
(2) Berberine vs. no treatment	2	206	−0.03 [−0.04, −0.02]	6.72	<0.001	0
Gastrointestinal adverse events	3	567	1.01 [0.76, 1.35]	0.08	0.94	0
(1) Berberine vs. placebo	3	567	1.01 [0.76, 1.35]	0.08	0.94	0
Serious events during pregnancy	1	430	0.98 [0.70, 1.37]	0.11	0.91	—
(1) Berberine vs. placebo	1	430	0.98 [0.70, 1.37]	0.11	0.91	—

*Berberine vs. metformin*						
Live birth	1	75	1.32 [0.78, 2.25]	1.02	0.31	—
Pregnancy	2	126	1.10 [0.69, 1.74]	0.39	0.70	0
Ovulation per subject	1	98	1.32 [1.03, 1.70]	2.18	0.03	—
TT	4	262	−0.10 [−0.17, −0.03]	2.75	0.01	0
SHBG	2	146	5.97 [1.02, 10.91]	2.37	0.02	0
FAI	2	146	−0.28 [−0.83, 0.28]	0.97	0.33	58
LH	4	262	−0.49 [−1.31, 0.33]	1.18	0.24	67
FSH	4	262	−0.17 [−0.79, 0.45]	0.55	0.58	82
LF	1	55	−0.90 [−1.58, −0.22]	2.59	0.01	—
FPG	4	262	−0.03 [−0.23, 0.16]	0.33	0.74	52
PPG	2	116	−0.13 [−0.51, 0.25]	0.69	0.49	0
FINS	4	262	−0.95 [−2.09, 0.20]	1.62	0.10	12
PPINS	1	55	2.39 [−31.93, 36.71]	0.14	0.89	—
HOMA	4	262	−0.22 [−0.47, 0.02]	1.78	0.08	0
TC	3	201	−0.44 [−0.60, −0.29]	5.61	<0.001	43
TG	3	201	0.02 [−0.19, 0.22]	0.14	0.89	82
HDL	4	262	0.05 [0.03, 0.08]	3.89	<0.001	45
LDL	4	262	−0.34 [−0.48, −0.21]	4.87	<0.001	0
BMI	4	262	−0.03 [−0.46, 0.39]	0.15	0.88	31
WC	3	197	−2.74 [−4.55, −0.93]	2.96	<0.001	33
WHR	4	258	−0.04 [−0.05, −0.03]	7.91	<0.001	25
Gastrointestinal adverse events	2	145	0.50 [0.23, 1.09]	1.75	0.08	21

*Berberine vs. letrozole*						
Live birth	1	429	0.61 [0.44, 0.82]	3.19	<0.001	—
Pregnancy	1	429	0.57 [0.43, 0.77]	3.63	<0.001	—
Conception	1	429	0.63 [0.48, 0.81]	3.57	<0.001	—
Ovulation per subject	1	429	0.79 [0.71, 0.87]	4.56	<0.001	—
Ovulation per cycle	1	1,627	0.61 [0.55, 0.68]	9.03	<0.001	—
Gastrointestinal adverse events	1	429	1.32 [1.00, 1.73]	1.97	0.05	—
Serious events during pregnancy	1	429	0.87 [0.61, 1.24]	0.78	0.44	—

*Z*-value: test for overall effect; *P* value: *P* value for *Z*-test; BMI: body mass index; WC: waist circumference; WHR: waist circumference-to-hip circumference ratio; TT: total testosterone; SHBG: sex-hormone binding globulin; FAI: free androgen index; LH: luteinizing hormone; FSH: follicle-stimulating hormone; LF: luteinizing hormone to follicle-stimulating hormone ratio; FPG: fasting plasma glucose; PPG: postprandial plasma glucose; FINS: fasting insulin; PPINS: postprandial plasma insulin; HOMA: homeostasis model assessment of insulin resistance; TC: total cholesterol; TG: triglycerides; HDL: high-density lipoprotein cholesterol; LDL: low-density lipoprotein cholesterol.

## Supplementary Materials

Supplementary Materials Supplementary Figure 1: assessment of bias risk of RCTs included in this systematic review and meta-analysis (: low risk of bias; : unclear risk of bias; : High: high risk of bias). Supplementary Figure 2: meta-analyses of the effect of berberine on reproductive index (A: total testosterone; B: sex hormone binding globulin; C: free androgen index; D: luteinizing hormone (LH); E: LH/FSH ratio) compared with placebo or no treatment. Supplementary Figure 3: meta-analyses of the effect of berberine on metabolic characteristics (A: fasting plasma glucose; B: fasting insulin; C: total cholesterol; D: triglyceride; E: HDL-C; F: LDL-C) compared with placebo or no treatment. Supplementary Figure 4: meta-analyses of the effect of berberine on waist-to-hip ratio compared with placebo or no treatment Supplementary Figure 5: meta-analyses of the effect of berberine on adiposis (A: waist circumference; B: waist-to-hip ratio) compared with metformin.

## References

[1] Azziz R., Carmina E., Chen Z. (2016). Polycystic ovary syndrome. *Nature Reviews Disease Primers*.

[2] Lizneva D., Suturina L., Walker W., Brakta S., Gavrilova-Jordan L., Azziz R. (2016). Criteria, prevalence, and phenotypes of polycystic ovary syndrome. *Fertility and Sterility*.

[3] Diamanti-Kandarakis E., Dunaif A. (2012). Insulin resistance and the polycystic ovary syndrome revisited: an update on mechanisms and implications. *Endocrine Reviews*.

[4] Brower M., Brennan K., Pall M., Azziz R. (2013). The severity of menstrual dysfunction as a predictor of insulin resistance in pcos. *The Journal of Clinical Endocrinology & Metabolism*.

[5] Legro R. S. (2016). Ovulation induction in polycystic ovary syndrome: current options. *Best Practice & Research Clinical Obstetrics & Gynaecology*.

[6] Penzias A., Bendikson K., Butts S. (2017). Role of metformin for ovulation induction in infertile patients with polycystic ovary syndrome (PCOS): a guideline. *Fertility and Sterility*.

[7] Bordewijk E. M., Nahuis M., Costello M. F. (2017). Metformin during ovulation induction with gonadotrophins followed by timed intercourse or intrauterine insemination for subfertility associated with polycystic ovary syndrome. *Cochrane Database of Systematic Reviews*.

[8] Morley L. C., Tang T., Yasmin E., Norman R. J., Balen A. H. (2017). Insulin-sensitising drugs (metformin, rosiglitazone, pioglitazone, D-chiro-inositol) for women with polycystic ovary syndrome, oligo amenorrhoea and subfertility. *The Cochrane Database of Systematic Reviews*.

[9] Abushouk A. I., Salem A. M. A., Abdel-Daim M. M. (2017). *Berberis vulgaris* for cardiovascular disorders: a scoping literature review. *Iranian Journal of Basic Medical Sciences*.

[10] Rahimi-Madiseh M., Lorigoini Z., Zamani-Gharaghoshi H., Rafieian-Kopaei M. (2017). *Berberis vulgaris*: specifications and traditional uses. *Iranian Journal of Basic Medical Sciences*.

[11] Imanshahidi M., Hosseinzadeh H. (2008). Pharmacological and therapeutic effects of Berberis vulgaris and its active constituent, berberine. *Phytotherapy Research*.

[12] Dong H., Wang N., Zhao L., Lu F. (2012). Berberine in the treatment of type 2 diabetes mellitus: a systemic review and meta-analysis. *Evidence-Based Complementary and Alternative Medicine*.

[13] Wang H., Zhu C., Ying Y., Luo L., Huang D., Luo Z. (2015). Metformin and berberine, two versatile drugs in treatment of common metabolic diseases. *Oncotarget*.

[14] Imenshahidi M., Hosseinzadeh H. (2019). Berberine and barberry (*Berberis vulgaris*): a clinical review. *Phytotherapy Research*.

[15] Wei W., Zhao H., Wang A. (2012). A clinical study on the short-term effect of berberine in comparison to metformin on the metabolic characteristics of women with polycystic ovary syndrome. *European Journal of Endocrinology*.

[16] Cheng Z., Pang T., Gu M. (2006). Berberine-stimulated glucose uptake in L6 myotubes involves both AMPK and p38 MAPK. *Biochimica et Biophysica Acta (BBA)—General Subjects*.

[17] Yin J., Hu R., Chen M. (2002). Effects of berberine on glucose metabolism in vitro. *Metabolism*.

[18] Turner N., Li J.-Y., Gosby A. (2008). Berberine and its more biologically available derivative, dihydroberberine, inhibit mitochondrial respiratory complex I: a mechanism for the action of berberine to activate AMP-activated protein kinase and improve insulin action. *Diabetes*.

[19] Lee Y. S., Kim W. S., Kim K. H. (2006). Berberine, a natural plant product, activates AMP-activated protein kinase with beneficial metabolic effects in diabetic and insulin-resistant states. *Diabetes*.

[20] Tao R., Gong J., Luo X. (2010). AMPK exerts dual regulatory effects on the PI3K pathway. *Journal of Molecular Signaling*.

[21] Gwirtn D. M., Shackelford D. B., Egan D. F. (2008). AMPK phosphorylation of raptor mediates a metabolic checkpoint. *Molecular Cell*.

[22] Wang M., Chen S., Zhang D. (2015). Effect of berberine versus metformin in women with polycystic ovary syndrome: a systematic review of randomized controlled trials. *Guangdong Medical Journal*.

[23] Chen X., Liu S., Han X. (2016). Effect of berberine in the treatment of polycystic ovary syndrome combined with insulin resistance. *Chinese Medicine and Pharmacy*.

[24] Li H., Yu J. (2016). Effect of Berberine combined with letrozole on ovulation induction in women with PCOS. *Journal of Practical Obstetrics and Gynecology*.

[25] Li X., Kuang H., Luo Y., Chen Q. (2017). Clinical observation of berberine in intervening insulin resistance of polycystic ovary syndrome. *Journal of Guangzhou University of Traditional Chinese Medicine*.

[26] Liu H., Deng L., Luo Y. (2017). Analysis of therapeutic effects of estradiol and progesterone combined with berberine on infertile patients with non obese polycystic ovary syndrome. *Jilin Medical Journal*.

[27] Wang P., Wang H., Wang Y. (2016). Clinical effect of metformin combined with berberine on obese women with polycystic ovary syndrome. *China’s Pprimary Health Care*.

[28] Wu X.-K., Wang Y.-Y., Liu J.-P. (2016). Randomized controlled trial of letrozole, berberine, or a combination for infertility in the polycystic ovary syndrome. *Fertility and Sterility*.

[29] Green S., Higgins P. J., Alderson T. P., Clarke M., Mulrow D. C., Oxman D. A. (2011). Assessing risk of bias in included studies. *Cochrane Handbook for Systematic Reviews of Interventions*.

[30] Zhu Q., Hu W., Dai C. (2016). Effects of berberine combined with ethinylestradiol cyproterone acetate in the treatment of obese women with polycystic ovary syndrome. *Chinese Journal of Primary Medicine and Pharmacy*.

[31] Ma Y., Yang J., Sui M., Liang K., Deng H., Wei W. (2011). Study the therapeutic effect of berberine on PCOS patients with insulin resistance. *Chinese Journal of Obstetrics and Gynecology*.

[32] Wang L., Kong Y., Ren Y., Shen M. (2011). Therapeutic effect of berberine combined with metformin for women with polycystic ovary syndrome and insulin resistance. *Journal of Zhejiang Chinese Medical University*.

[33] An Y., Sun Z., Zhang Y., Liu B., Guan Y., Lu M. (2014). The use of berberine for women with polycystic ovary syndrome undergoing IVF treatment. *Clinical Endocrinology*.

[34] Liu W. (2015). *The Clinical Study on the Effect of Berberine Combined with Cang-Fu-Dao-Tan Decoction Treating Obese Women with Polycystic Ovary Syndrom*.

[35] Azziz R., Tarlatzis R., Dunaif A. (2004). Revised 2003 consensus on diagnostic criteria and long-term health risks related to polycystic ovary syndrome. *Fertility and Sterility*.

[36] Diamond M. P., Carson S. A., Steinkampf M. P. (2007). Clomiphen, metformin, or both for infertility in the polycystic ovary syndrome. *The New England Journal of Medicine*.

[37] Micic D., Popovic V., Nesovic M. (1988). Androgen levels during sequential insulin euglycemic clamp studies in patients with polycystic ovary disease. *Journal of Steroid Biochemistry*.

[38] Burghen G. A., Givens J. R., Kitabchi A. E. (1980). Correlation of hyperandrogenism with hyperinsulinism in polycystic ovarian disease. *The Journal of Clinical Endocrinology & Metabolism*.

[39] Nestler J. E., Jakubowicz D. J., De Vargas A. F., Brik C., Quintero N., Medina F. (1998). Insulin stimulates testosterone biosynthesis by human thecal cells from women with polycystic ovary syndrome by activating its own receptor and using inositolglycan mediators as the signal transduction system. *Journal of Clinical Endocrinology & Metabolism*.

[40] Munir I., Yen H.-W., Geller D. H. (2004). Insulin augmentation of 17*α*-hydroxylase activity is mediated by phosphatidyl inositol 3-kinase but not extracellular signal-regulated kinase-1/2 in human ovarian theca cells. *Endocrinology*.

[41] Barbieri R. L., Makris A., Ryan K. J. (1983). Effects of insulin on steroidogenesis in cultured porcine ovarian theca. *Fertility and Sterility*.

[42] Franks S., Gilling-Smith C., Watson H., Willis D. (1999). Insulin action in the normal and polycystic ovary. *Endocrinology and Metabolism Clinics of North America*.

[43] Rice S., Christoforidis N., Gadd C. (2005). Impaired insulin-dependent glucose metabolism in granulosa-lutein cells from anovulatory women with polycystic ovaries. *Human Reproduction*.

[44] Willis D. S., Watson H., Mason H. D., Galea R., Brincat M., Franks S. (1998). Premature response to luteinizing hormone of granulosa cells from anovulatory women with polycystic ovary syndrome: relevance to mechanism of anovulation. *Journal of Clinical Endocrinology & Metabolism*.

[45] Wu X., Yao J., Hou L., Kuang H. (2006). P-876: berberine improves insulin resistance in granulosa cells in a similar way to metformin. *Fertility and Sterility*.

[46] Zhao L., Li W., Han F. (2011). Berberine reduces insulin resistance induced by dexamethasone in theca cells in vitro. *Fertility and Sterility*.

[47] Adashi E. Y., Hsueh A. J. W., Yen S. S. C. (1981). Insulin enhancement of luteinizing hormone and follicle-stimulating hormone release by cultured pituitary cells. *Endocrinology*.

[48] Brothers K. J., Wu S., Divall S. A. (2010). Rescue of obesity-induced infertility in female mice due to a pituitary-specific knockout of the insulin receptor. *Cell Metabolism*.

[49] Vuddanda P. R., Chakraborty S., Singh S. (2010). Berberine: a potential phytochemical with multispectrum therapeutic activities. *Expert Opinion on Investigational Drugs*.

[50] Yin J., Xing H., Ye J. (2008). Efficacy of berberine in patients with type 2 diabetes mellitus. *Metabolism*.

[51] Zhang Y., Li X., Zou D. (2008). Treatment of type 2 diabetes and dyslipidemia with the natural plant alkaloid berberine. *The Journal of Clinical Endocrinology & Metabolism*.

[52] Kong W., Wei J., Abidi P. (2004). Berberine is a novel cholesterol-lowering drug working through a unique mechanism distinct from statins. *Nature Medicine*.

[53] Dong H., Zhao Y., Zhao L., Lu F. (2013). The effects of berberine on blood lipids: a systemic review and meta-analysis of randomized controlled trials. *Planta Medica*.

[54] Li M. F., Zhou X. M., Li X. L. (2018). The effect of berberine on polycystic ovary syndrome patients with insulin resistance (PCOS-IR): a meta-analysis and systematic review. *Evidence-Based Complementary and Alternative Medicine*.

[55] Xia X., Yan J., Shen Y. (2011). Berberine improves glucose metabolism in diabetic rats by inhibition of hepatic gluconeogenesis. *PLoS One*.

[56] Yin J., Gao Z., Liu D., Liu Z., Ye J. (2008). Berberine improves glucose metabolism through induction of glycolysis. *American Journal of Physiology-Endocrinology and Metabolism*.

[57] Zhou L., Yang Y., Wang X. (2007). Berberine stimulates glucose transport through a mechanism distinct from insulin. *Metabolism*.

[58] La Marca A., Grisendi V., Dondi G., Sighinolfi G., Cianci A. (2015). The menstrual cycle regularization following D-chiro-inositol treatment in PCOS women: a retrospective study. *Gynecological Endocrinology*.

[59] Cianci A., Panella M., Fichera M., Falduzzi C., Bartolo M., Caruso S. (2015). d-chiro-Inositol and alpha lipoic acid treatment of metabolic and menses disorders in women with PCOS. *Gynecological Endocrinology*.

[60] Chen Y., Li Y., Wang Y., Wen Y., Sun C. (2009). Berberine improves free-fatty-acid- induced insulin resistance in L6 myotubes through inhibiting peroxisome proliferator-activated receptor *γ* and fatty acid transferase expressions. *Metabolism*.

[61] Abidi P., Zhou Y., Jiang J.-D., Liu J. (2005). Extracellular signal-regulated kinase-dependent stabilization of hepatic low-density lipoprotein receptor mRNA by herbal medicine berberine. *Arteriosclerosis, Thrombosis, and Vascular Biology*.

[62] Brusq J.-M., Ancellin N., Grondin P. (2006). Inhibition of lipid synthesis through activation of AMP kinase: an additional mechanism for the hypolipidemic effects of berberine. *Journal of Lipid Research*.

[63] Derosa G., D’Angelo A., Bonaventura A., Bianchi L., Romano D., Maffioli P. (2013). Effects of berberine on lipid profile in subjects with low cardiovascular risk. *Expert Opinion on Biological Therapy*.

[64] Zuo F., Nakamura N., Akao T., Hattori M. (2006). Pharmacokinetics of berberine and its main metabolites in conventional and pseudo germ-free rats determined by liquid chromatography/ion trap mass spectrometry. *Drug Metabolism and Disposition*.

