# Supplementary material for: The Effect of Berberine on Reproduction and Metabolism in Women with Polycystic Ovary Syndrome: A Systematic Review and Meta-Analysis of Randomized Control Trials
Source: Evid Based Complement Alternat Med. 2019 Dec 13;2019:7918631. doi: 10.1155/2019/7918631 (PMC6930782; doi:10.1155/2019/7918631)
Supplement: Supplementary Materials — Supplementary Figure 1: assessment of bias risk of RCTs included in this systematic review and meta-analysis (: low risk of bias; : unclear risk of bias; : High: high risk of bias). Supplementary Figure 2: meta-analyses of the effect of berberine on reproductive index (A: total testosterone; B: sex hormone binding globulin; C: free androgen index; D: luteinizing hormone (LH); E: LH/FSH ratio) compared with placebo or no treatment. Supplementary Figure 3: meta-analyses of the effect of berberine on metabolic characteristics (A: fasting plasma glucose; B: fasting insulin; C: total cholesterol; D: triglyceride; E: HDL-C; F: LDL-C) compared with placebo or no treatment. Supplementary Figure 4: meta-analyses of the effect of berberine on waist-to-hip ratio compared with placebo or no treatment Supplementary Figure 5: meta-analyses of the effect of berberine on adiposis (A: waist circumference; B: waist-to-hip ratio) compared with metformin. [file 7918631.f1.docx]

***Supplementary figure 1 Assessment of bias risk of RCTs included in this systematic review and meta-analysis.***


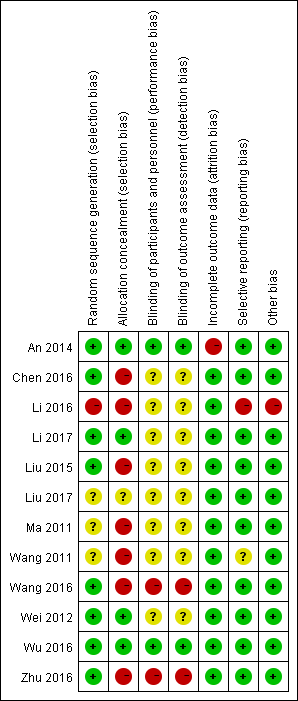


Note:
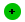
: low risk of bias;
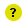
: unclear risk of bias;
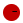
:High: high risk of bias.

***SupplementaryFigure 2***


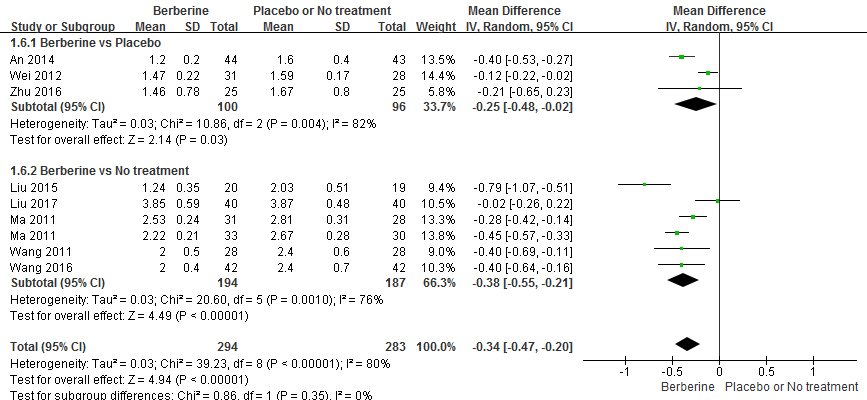
A


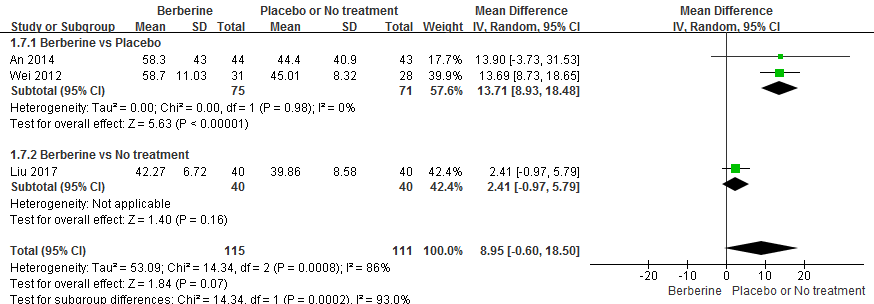
B


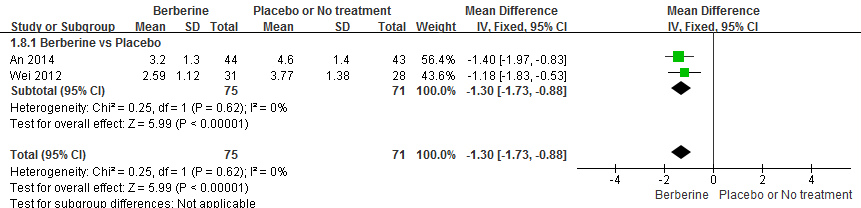
C


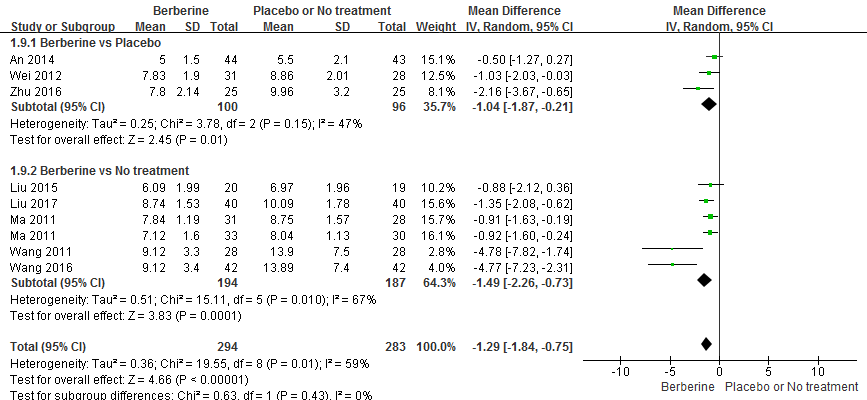
D


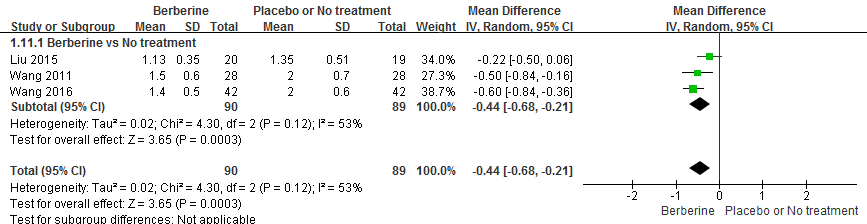
E

Meta-analyses of the effect of berberine on reproductive index (A: Total Testosterone; B: Sex Hormone Binding Globulin; C: Free androgen index; D: Luteinizing Hormone (LH); E: LH/FSH ratio) compared with placebo or no treatment.

***SupplementaryFigure 3***


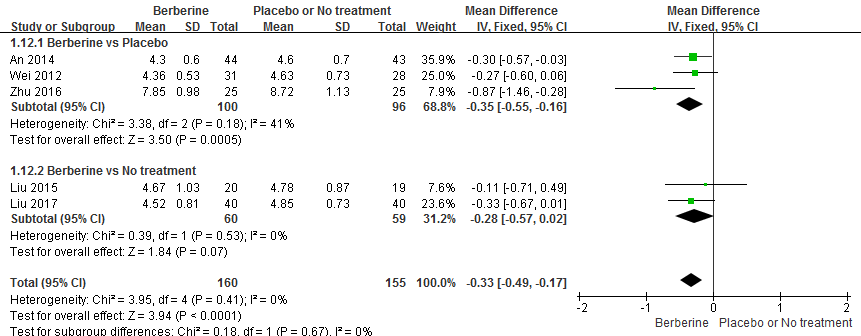
A

B


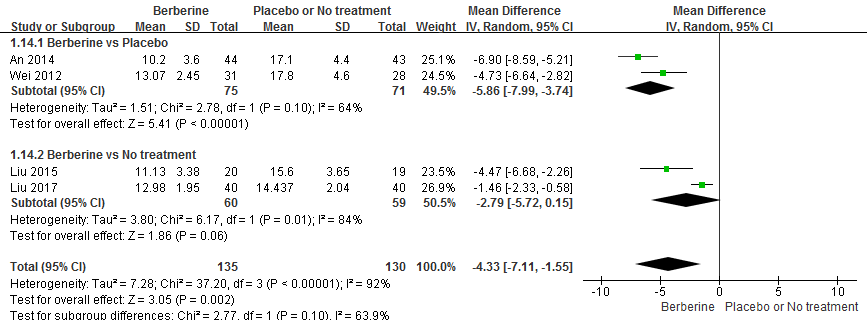


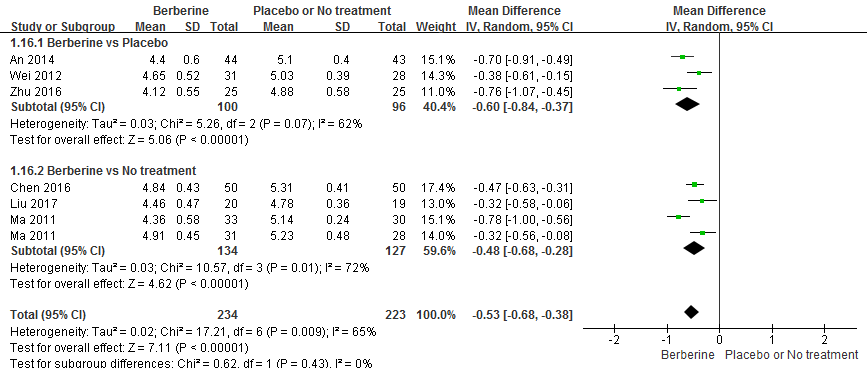
C


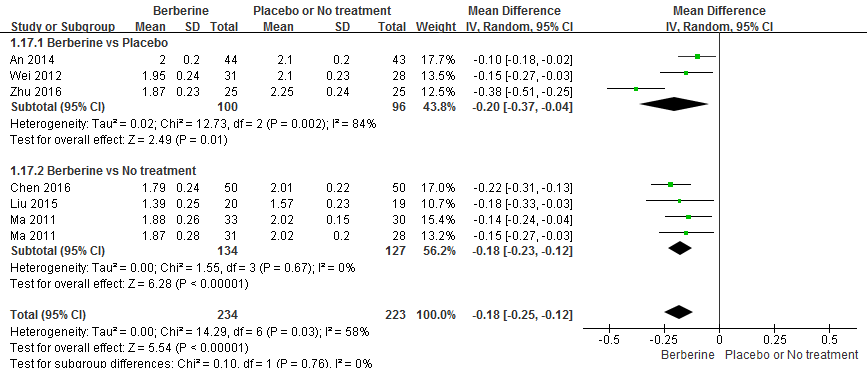
D


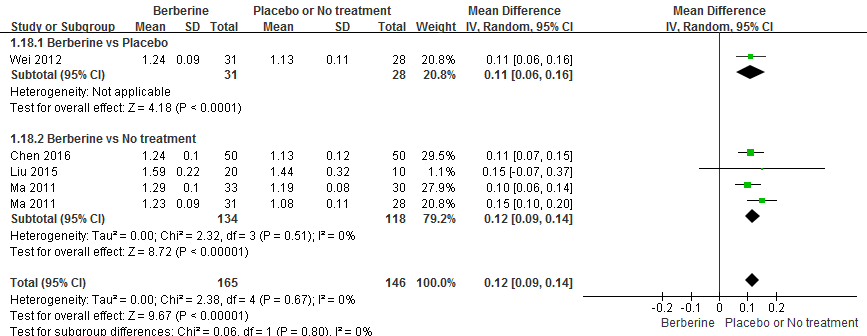
E


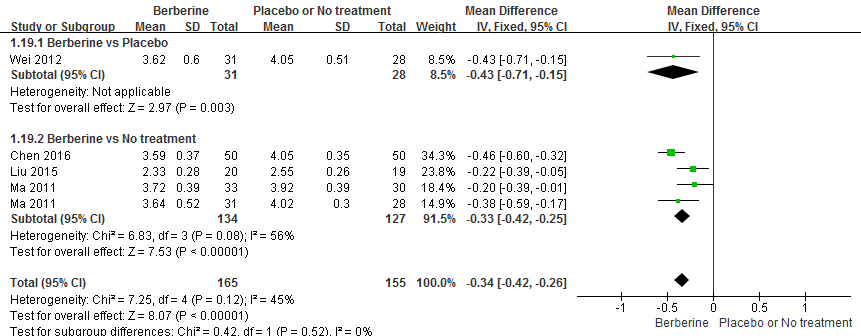
F

Meta-analyses of the effect of berberine on metabolic characteristics (A: Fasting plasma glucose; B: Fasting insulin; C: Total cholesterol; D: Triglyceride; E: HDL-C; F: LDL-C) compared with placebo or no treatment.


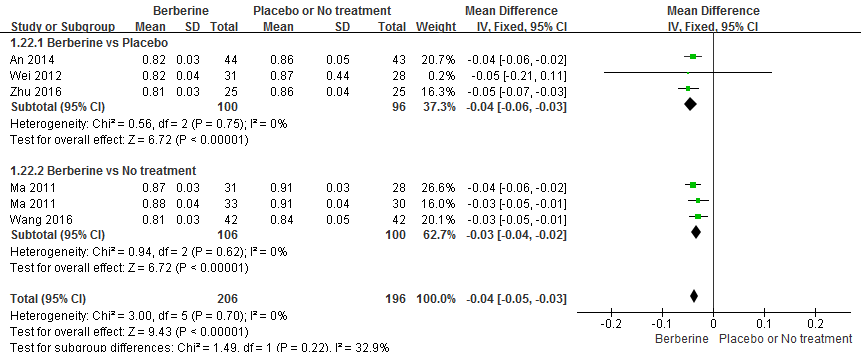
***Supplementary Figure 4***

Meta-analyses of the effect of berberine on waist to hip ratio compared with placebo or no treatment.

***SupplementaryFigure 5***


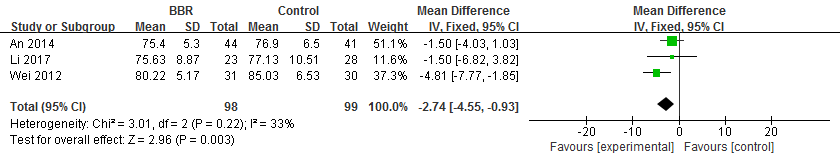
A


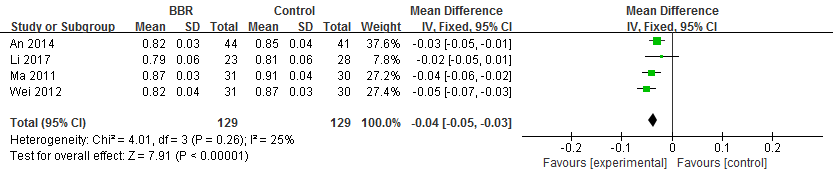
B

Meta-analyses of the effect of berberine on adiposis (A: Waist circumference; B: Waist to hip ratio) compared with metformin.
